# Clinical Cases

**Published:** 1883-10

**Authors:** E. Rushmore

**Affiliations:** Plainfield, New Jersey


### CLINICAL CASES.

E. Rushmore, M. D., Plainfield, New Jersey.

*Read before the New Jersey State Homœopathic Medical Society.*

Case I.—Mr. S., of middle age. Early this spring I was asked to visit him in haste, and found him tossing around on the bed, with screams from the violence of abdominal pain, to attacks of which he is subject at intervals of a few months. He could not speak much to describe his pain, but said it was sharp, having paroxysms of increased severity, when it would shoot down the legs from the abdomen. The abdomen was hard and much retracted. No stool for several days. Had taken enemas and applied mustard and heat to the abdomen without relief.

Many remedies have pain in the lower limbs with abdominal pain, but my manuscript repertory, which I generally carry, gave only Kali carb. as having pain extending from the stomach to the lower limbs, and in this case the pains were also of the peculiar shooting character of Kali carbonicum. A few globules of Fincke's 900th potency were dropped in half a tumblerful of water, and as soon as dissolved a teaspoonful of the solution was administered to the patient. In five minutes there was little change and the dose was repeated, but within fifteen minutes the relief was sensible and progressing, and in half an hour he was quite free from pain. I had not removed the dry heat from the abdomen. Then came a desire for stool, and an enema of warm water, as he said the stools were always very hard, was administered, and he had a free evacuation. The next day he felt as well as usual, except a little soreness.

For these attacks he had formerly taken Morphia, after which he said he would be sick for several days. Was this relief a recovery or a cure? The only thing I did before he got relief was to administer the chosen similar remedy; after the pain was relieved followed the much needed alvine evacuation, and this order of occurrences we are most reasonably assured is the sign of a cure. We have additional evidence that the relief was due to the medicine in the fact that in his last former, but less severe, similar attack the administration of the same remedy in the CM potency, seemed to act equally well.

Case II.—August 14th, 1882. Mrs. B has steadily pain in the back, worse from sitting and lying, and relieved by walking; it feels like a pressure in the epigastrium extending to the back. There is a heavy pain in the abdomen with the pain in the back. Also headache, beginning in the afternoon in the nape of the neck, with sleep, which relieves. She goes three days without stool, or any desire for it.

Guided by Lippe's Repertory, I was led to the selection of Strontium carbonicum as meeting best of all this peculiar combination of conditions and concomitants. Headache beginning in the afternoon or evening, attended with sleepiness, and backache, worse when sitting, but relieved when walking, with pressure in the abdomen, and with constipation, were together found only under the Strontium. It was given in the 200th attenuation every evening and morning for three days. In three days the report was of better stool, no pain in the back, and headache only one day. She got no more medicine, and in nine days reported having regular stools and being better every way.

Case III.—November 2d, 1882. Mr. T. has stitches up the back, relieved by drawing the shoulders backward; flashes of heat in the face; bad taste; cough worse from stooping and expectoration. My manuscript repertory gave under stitches *upward* in back, Cyclamen and Staphysagria; of these only Cyclamen had relief from drawing back the shoulders. Cyclamen also had the bad taste, the heat in face, and cough. He received Cyclamen$^{2c}$, to dose every four hours till better. The next day he called to say that he felt like a new man, had almost no cough, and was entirely free from pain in the back.

Case IV.—March 21st, 1883. Mr. C., who had for several weeks occasionally spoken with me about his case, and seemed to have been prevented from sooner placing himself under my care by my refusal to promise a permanent cure in a few days, has concluded to try homœopathic treatment. He suffers now from intermittent fever, has had it summer and winter for two years. The type is tertian; the attacks begin with gaping, stretching, lachrymation, and pain in the bones. The chill lasts two or three hours, and with it he has thirst in the beginning, chattering of teeth, blue nails, and vomiting of sour food near the end. The heat lasts two or three hours more; with it he has bursting headache, thirst, and vomiting from drinking. After the heat there is sleep with weakness. There is no mention of sweat. He received at once one dose dry of Arsenicum $50^{m}$ (Skinner), and another to be taken three hours before he expected the next chill. Although he takes much medicine, he loses several days from work every week with his chills. In ten days he reported no chill since taking the medicine, but he still has the gaping and profuse lachrymation at the usual chill time. On account of the gaping and lachrymation, he then got Rhus tox.$^{cm}$ (F. C.), one dose, and at the end of a week said he had no lachrymation or gaping till that day, and had not lost a day from his work, neither had he yet had any chill.

